# The Neutrophil-to-Lymphocyte Ratio Is an Important Indicator Predicting In-Hospital Death in AMI Patients

**DOI:** 10.3389/fcvm.2021.706852

**Published:** 2021-09-20

**Authors:** Zhenjun Ji, Guiren Liu, Jiaqi Guo, Rui Zhang, Yamin Su, Abdlay Carvalho, Yangyang Qu, Wenjie Zuo, Yuyu Yao, Jie Lin, Genshan Ma

**Affiliations:** ^1^Department of Cardiology, Zhongda Hospital, School of Medicine, Southeast University, Nanjing, China; ^2^Department of Epidemiology and Health Statistics, School of Public Health, Southeast University, Nanjing, China; ^3^Department of Cardiology, Jiangsu Taizhou People's Hospital, The Fifth Affiliated Hospital of Nantong University, Taizhou, China

**Keywords:** neutrophil to lymphocyte ratio, acute myocardial infarction (AMI), biomarker, blood routine examination, prognosis

## Abstract

**Objective:** To explore the role of neutrophil-to-lymphocyte ratio (NLR) in predicting the short-term prognosis of NSTEMI and STEMI.

**Methods:** This study was a single-center, retrospective and observational study. 2618 patients including 1289 NSTMI and 1329 STEMI patients were enrolled from June 2013 to February 2018 in Zhongda Hospital, Southeast University. The demographic information, clinical characteristics, medical history, laboratory examination, treatment, and outcome of individuals at admission and during hospitalization were extracted from the electronic medical record system. Outcome was defined as the all-cause death during hospitalization.

**Results:** (1) In the NSTEMI group, the ability of NLR in predicting in-hospital death (AUC = 0.746) was higher than the neutrophil-monocyte ratio (NMR) (AUC = 0.654), the platelet-lymphocyte ratio (PLR) (AUC = 0.603) and the lymphocyte-monocyte ratio (LMR) (AUC = 0.685), and also higher than AST (AUC = 0.621), CK (AUC = 0.595), LDH (AUC = 0.653) and TnI (AUC = 0.594). The AUC of NLR in the STEMI group was only 0.621. (2) The optimal cut-off value of NLR in NSTEMI group was 5.509 (Youden index = 0.447, sensitivity = 77.01%, specificity = 67.72%). After adjusting variables including age, sex, diabetes history, smoking history, LDL-C and Cr, the logistic regression showed that the patients with NLR>5.509 had higher hazard risk of death (HR4.356; 95%CI 2.552–7.435; *P* < 0.001) than the patients with NLR ≤ 5.509. (3) Stratification analysis showed that the in-hospital mortality of patients with NLR > 5.509 was 14.611-fold higher than those with NLR ≤ 5.509 in patients aged <76, much higher than the ratio in patients aged ≥ 76. For patients with creatinine levels ≤ 71, the in-hospital death risk in high NLR group was 10.065-fold higher than in low NLR group (95%CI 1.761–57.514, *P* = 0.009), while the HR was only 4.117 in patients with creatinine levels > 71. The HR in patients with or without diabetes were 6.586 and 3.375, respectively. The HR in smoking or no smoking patients were 6.646 and 4.145, respectively. The HR in patients with LDL-C ≥ 2.06 or <2.06 were 5.526 and 2.967 respectively.

**Conclusion:** Compared to NMR, PLR, and LMR, NLR had the best ability in predicting in-hospital death after NSTEMI. Age, creatinine, LDL-C, diabetes and smoking history were all important factors affecting the predictive efficiency in NSTEMI. NLR had the limited predictive ability in STEMI.

## Introduction

Acute myocardial infarction (AMI) is a serious and fatal cardiovascular emergency (1). Rupture of vulnerable coronary plaques and thrombosis formation can result in complete or partial coronary artery occlusion and myocardial ischemia or necrosis, which is an important pathophysiological process of myocardial infarction. Pathological features of vulnerable plaques are thin-cap fibroatheroma (TCFA ≤ 65 μm), large lipid pool, inflammation in vessels (macrophages/mononuclear cell infiltration), and intimal erosion with platelet aggregation, plaques rupture, superficial calcification (2, 3). Inflammation is an important mechanism for the rupture of vulnerable plaques. A large number of inflammatory cells, mainly neutrophils, infiltrate the coronary artery atheromatous plaque, and gather in the vessel wall, promoting vascular endothelial cells adhesion and activation, and secreting peroxidase and tissue damage factor such as alkaline phosphatase (4). Neutrophils and lymphocytes-mediated immune response both can cause rupture of coronary plaque (4). The monocyte-macrophage system also produces various proteases (collagenase, matrix lysozyme, etc.) digesting the fibrous cap, making the thin fibrous cap more unstable.

Neutrophil-lymphocyte ratio (NLR), refers to the absolute ratio of peripheral blood neutrophils to lymphocytes, which can reflect the state of systematic inflammation. Compared with other leukocyte subtypes and inflammatory markers, NLR is easier to obtain from stable and reliable blood routine examination (BRE). They are closely related to the severity and prognosis of cardiovascular diseases (5), infectious diseases (6), malignant tumors (7) and other diseases. In addition, there are many indicators related to neutrophils and lymphocytes. The neutrophil-monocyte ratio (NMR) (8), the platelet-lymphocyte ratio (PLR) (9) and the lymphocyte-monocyte ratio (LMR) (10) were all important inflammation indicators for predicting adverse outcome in inflammatory diseases.

Here, this study aimed to explore the role of NLR in predicting the short-term prognosis of non–ST-segment elevation myocardial infarction (NSTEMI) and ST-segment elevation myocardial infarction (STEMI), and compare its predictive performance with other common indicators, such as NMR, PLR and LMR.

## Methods

### Study Participants

This study was a single-center, retrospective and observational study, approved by the Ethics Committee of Zhongda Hospital affiliated to Southeast University (2020ZDSYLL164-P01). In total, 2618 patients including 1289 NSTMI and 1329 STEMI patients admitted to the Zhongda hospital affiliated to Southeast University from June 2013 to February 2018 were enrolled in this study. Inclusion criteria: 1. Age > 18; 2. Diagnosed as STEMI or NSTEMI. Exclusion criteria: 1. Suffering from severe diseases (such as late malignant tumor) with a life expectancy of less than half a year; 2. Pregnant or lactating women.

### Data Collection and Imputation

Demographic information, clinical characteristics, medical history, laboratory examination, treatment, and outcome of individuals at admission and during hospitalization were extracted from an electronic medical record system (By Yidu Cloud system). Medical histories such as hypertension, Type 2 diabetes and chronic kidney disease were all recorded. Results of the first laboratory examination after admission were extracted, including blood routine test, biochemical test for liver and kidney function, and blood coagulation function. Individuals' medications during hospitalization were also analyzed, including oral antidiabetic agents, antihypertensive drugs, lipid-lowering drugs, β-blockers, diuretics, aspirin. Grace score was calculated by the GRACE 2.0 ACS risk calculator. Outcome was defined as the all-cause death during hospitalization. Data was confirmed by experienced physicians and statisticians.

The missing data imputation was performed by R 3.5.1 software (11) (Supplementary Tables 1, 2). Firstly, variables with excessive missing values (>50%) were excluded, and then random forest multiple interpolation (RFMICE) by chain equation (five iterations) was used for data imputation. Finally, the average value of the five iterations was used as the final filling data. Baseline data before and after imputation were compared to determine the reliability of imputed data.

### Statistical Analysis

Data were processed by SPSS 23.0, MedCalc 15.0 and GraphPad Prism 7.0. For categorical variables, data was described as frequency or percentage. For continuous variables, if they conform to normal distribution, data was presented as mean ± standard deviation, otherwise, data was presented as quartiles [median (quartile 25, 75%)]. If continuous data satisfied normality, comparison between two groups or among multiple groups was analyzed by *t*-test or ANOVA analysis, otherwise, non-parametric test was used. Fisher's exact or Chi-square test were used for comparison of categorical variables. Univariate and variate logistic regression were performed for determining the hazard ratio (HR) of blood cell variables. Receiver operating characteristic (ROC) curve was used for analyzing the cut-off value of NLR and comparing efficiencies of different ratios. *P* < 0.05 was considered statistically significant.

## Results

### Baseline Characteristic of the Subjects

The baseline characteristic of the study cohort was presented in Table 1 and Supplementary Table 3. In total, 2,618 patients were enrolled in this retrospective study. Among the 1,329 STEMI patients and 1289 NSTEMI patients, 106 and 87 all-cause death were, respectively, recorded during hospitalization. The mean values of age in STEMI patients with or without events were 79.67 ± 11.47 and 68.17 ± 13.70, respectively, while the average age in NSTEMI patients with or without events were 84.47 ± 6.86 and 73.33 ± 12.69 respectively. Compared with the AMI group without death, the levels of white blood cells (WBC), neutrophils, uric acid (UA), cystatin C (CysC) and glucose (GLU) were all increased in the AMI death group (*P* < 0.05). In contrast, the levels of lymphocytes, hemoglobin (Hb) and albumin (ALB) were all decreased in the AMI death group (*P* < 0.05).

**Table 1:** Basic characteristics of enrolling patients.

**Characteristics**	**STEMI**		***P*-value**	**NSTEMI**		***P*-value**
	**Without death (*n* = 1,223)**	**With death (*n* = 106)**		**Without death (*n* = 1,202)**	**With death (*n* = 87)**	
Sex (male/female)	942(281)	71(35)	0.020*	820(382)	43(44)	<0.001***
Smoking	1,223(594)	106(32)	<0.001***	1,202(445)	87(16)	<0.001***
Age (years)	68.17 ± 13.70	79.67 ± 11.47	<0.001***	73.33 ± 12.69	84.47 ± 6.86	<0.001***
SBP (mmHg)	128.58 ± 21.82	119.00 ± 27.53	0.002**	137.08 ± 0.950	125.33 ± 26.36	0.001**
**Blood routine examination**						
Neutrophils (10^9^/L)	8.13 ± 4.15	10.02 ± 5.72	0.001**	6.26 ± 3.45	9.84 ± 6.08	<0.001***
Monocytes (10^9^/L)	0.46(0.34, 0.66)	0.57(0.40, 0.83)	<0.001***	0.48 ± 0.24	0.58 ± 0.36	0.014*
Lymphocytes (10^9^/L)	1.43 ± 0.72	1.24 ± 0.69	0.011*	1.42 ± 0.66	1.11 ± 0.64	<0.001***
WBC (10^9^/L)	10.23 ± 4.50	11.99 ± 6.00	0.004**	8.32 ± 3.60	11.58 ± 6.23	<0.001***
MCV (fL)	89.71 ± 5.17	89.68 ± 6.39	0.973	90.17 ± 5.57	90.71 ± 7.97	0.531
MCH (pg)	30.23 ± 1.99	29.76 ± 2.34	0.046*	30.11 ± 2.22	29.80 ± 2.85	0.318
MCHC (g/L)	336.98 ± 11.48	331.33 ± 14.33	<0.001***	333.78 ± 12.79	328.82 ± 15.13	0.004**
MPV (fL)	10.63 ± 1.32	10.92 ± 1.39	0.030*	10.73 ± 1.38	10.93 ± 1.49	0.180
Hct (%)	39.32 ± 5.75	36.17 ± 7.56	<0.001***	38.38 ± 6.44	32.96 ± 7.28	<0.001***
PLT (10^9^/L)	206.10 ± 65.09	189.19 ± 82.56	0.042*	194.57 ± 67.71	179.47 ± 75.28	0.046*
Hb (g/L)	132.59 ± 20.68	120.69 ± 26.31	<0.001***	128.40 ± 23.20	109.38 ± 26.11	<0.001***
**Biochemical test**						
ALT (U/L)	37(23, 59)	34.50(16.00, 70.75)	0.822	23(15, 34)	29(13, 44)	0.279
AST (U/L)	89(33, 191)	70.50(29.00, 211.50)	0.489	30(20, 57)	45(23, 118)	0.147
LDH (U/L)	419(250, 717)	427.00(247.25, 809.75)	0.623	232(178, 319)	307(222, 553)	0.003**
LDLC (mmol/L)	2.78 ± 0.89	2.48 ± 0.97	0.001**	2.69 ± 0.90	2.35 ± 0.87	0.001**
HDLC (mmol/L)	1.09 ± 0.26	1.00 ± 0.22	0.001**	1.08 ± 0.27	1.03 ± 0.31	0.131
BUN (mmol/L)	5.40(4.40, 7.20)	7.35(5.35, 12.83)	<0.001***	7.74 ± 5.63	13.83 ± 8.33	<0.001***
UA (μmol/L)	347.44 ± 114.88	401.51 ± 205.69	0.009**	360.37 ± 127.75	401.39 ± 145.23	0.012*
TnI (ng/ml)	3.54(0.57, 12.00)	2.59(0.33, 11.50)	0.648	0.685(0.14, 2.65)	1.6(0.25, 8.90)	0.003**
TC (mmol/L)	4.47 ± 1.17	4.03 ± 1.18	<0.001***	4.36 ± 1.19	4.00 ± 1.16	0.006**
ALB (g/L)	37.24 ± 4.79	34.09 ± 5.86	<0.001***	37.03 ± 5.08	33.21 ± 6.08	<0.001***
Creatinine (μmol/L)	101.35 ± 97.89	150.67 ± 124.70	<0.001***	88(70, 115)	140(104, 209)	<0.001***
CK (U/L)	654(134, 1768)	434.50(99.00, 1390.50)	0.122	141(77, 335)	265(95, 513)	0.029*
GLU (mmol/L)	8.32 ± 3.90	10.66 ± 6.54	<0.001***	8.29 ± 4.44	10.11 ± 5.94	0.006**

If the continuous data fits a normal distribution, it was described by X ± SD, otherwise, it was described by median and quartile (25%, 75%). *P < 0.05; **P < 0.01; ***P < 0.001. SBP, systolic blood pressure; ALT, alamine aminotransferase; AST, aspartate aminotransferase; LDH, lactate dehydrogenase; LDL-C, low-density lipoprotein cholesterol; HDL-C, high-density lipoprotein cholesterol; BUN, blood urea nitrogen; UA, uric acid; TC, total cholesterol; ALB, albumin; CK, creatine kinase; GLU, glucose; WBC, white blood cells; MCV, mean corpusular volume; MCH, mean corpsular hemoglobin; MCHC, mean corpsular hemoglobin concentration; MPV, mean platelet volume; Hct, hematocrit; Hb, hemoglobin.

### Blood Cell Counts Are Associated With Adverse Outcome Both in NSTEMI and STEMI Patients

In this study, we further analyzed the hazard ratio of indicators in BRE in AMI patients by univariate logistic regression (Table 2). Neutrophil (STEMI: HR1.08; 95%CI 1.04–1.12; *P* < 0.001 vs. NSTEMI: HR1.16; 95%CI 1.12–1.21; *P* < 0.001) and monocyte counts (STEMI: HR1.06; 95%CI 0.94–1.19; *P* = 0.347 vs. NSTEMI: HR3.37; 95%CI 1.69–6.73; *P* = 0.001) were the top two factors positively associated with adverse events in both STEMI and NSTEMI groups. Lymphocyte count was the main factor negatively associated with the adverse outcome in STEMI (HR0.65; 95%CI 0.46–0.90; *P* = 0.011) and NSTEMI (HR0.40; 95%CI 0.26–0.61; *P* = 0.000) groups.

**Table 2:** Univariate logistic regression of blood cell counts predicting in-hospital death in STEMI and NSTEMI patients.

**Parameters**	**STEMI**			**NSTEMI**		
	**HR**	**95%CI**	***P*-value**	**HR**	**95%CI**	***P*-value**
WBC (10^9^/L)	1.06	1.03–1.10	0.000	1.14	1.10–1.19	<0.001***
Neutrophil (10^9^/L)	1.08	1.04–1.12	0.000	1.16	1.12–1.21	<0.001***
Lymphocyte (10^9^/L)	0.65	0.46–0.90	0.011	0.40	0.26–0.61	<0.001***
Monocyte (10^9^/L)	1.06	0.94–1.19	0.347	3.37	1.69–6.73	0.001**
Platelet (10^9^/L)	1.00	0.99–1.00	0.012	1.00	0.99–1.00	0.044*
Hemoglobin (g/L)	0.98	0.97–0.99	0.000	0.97	0.96–0.98	<0.001***

*P < 0.05; **P < 0.01; ***P < 0.001. WBC, white blood cells.

**Figure 1:**
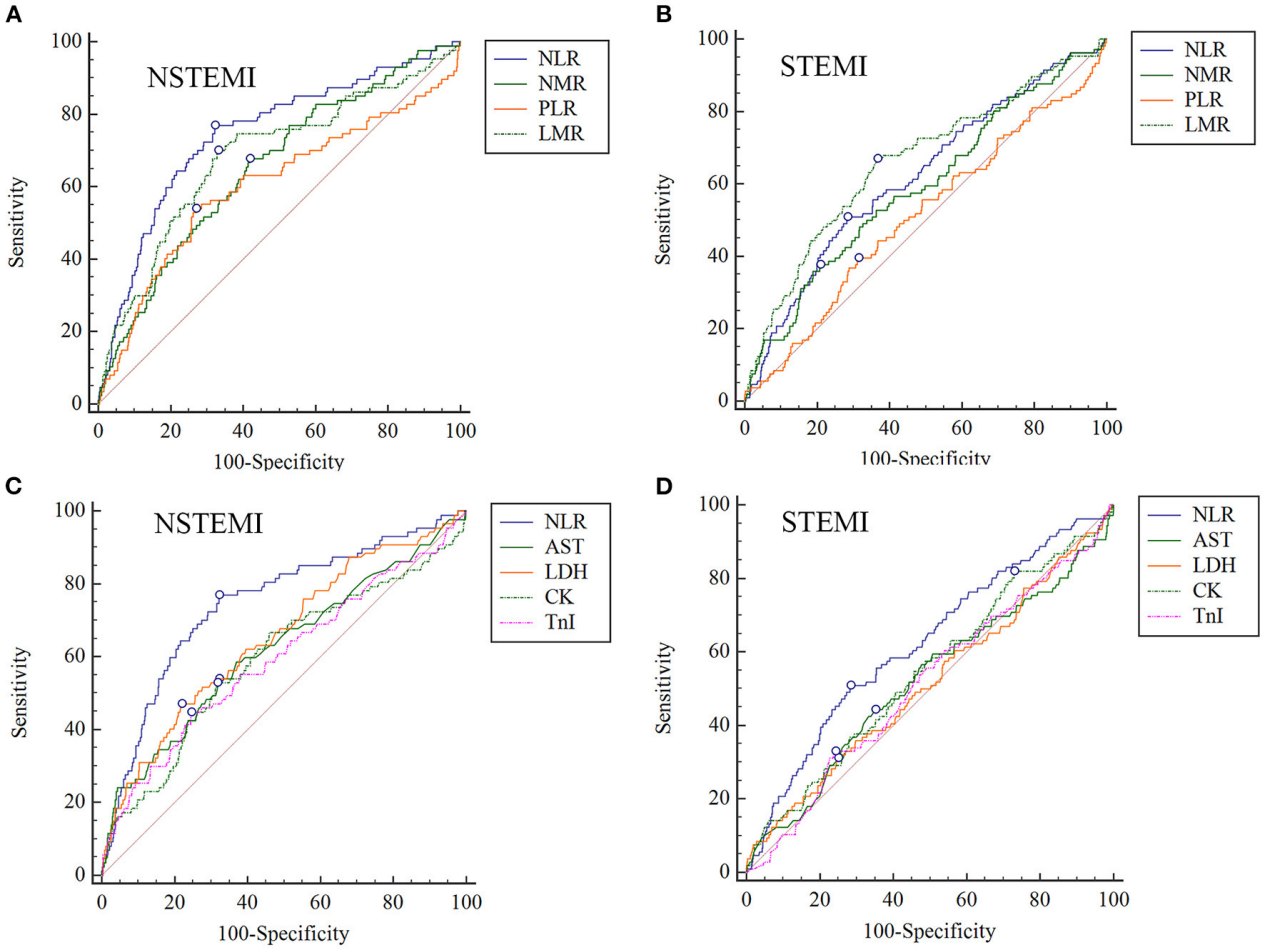
Comparison of ROC curves of common ratios and biomarkers in NSTEMI and STEMI groups, respectively. **(A,B)** Comparison of NLR, NMR, PLR, and LMR in predicting in-hospital death in NSTEMI and STEMI groups, respectively. **(C,D)** Comparison of NLR, AST, LDH, CK, and TnI in NSTEMI and STEMI groups, respectively. NLR, neutrophil-to-lymphocyte ratio; NMR, neutrophil-monocyte ratio; PLR, platelet-lymphocyte ratio; LMR, lymphocyte-monocyte ratio; AST, aspartate aminotransferase; LDH, lactate dehydrogenase; CK, creatine kinase.

Here, we also respectively studied the roles of these indicators in STEMI and NSTEMI. The ROC curve showed that NLR has the maximum area under the curve (AUC) in NSTEMI (AUC = 0.746, *P* < 0.0001) group, much higher than NMR (AUC = 0.654, *P* < 0.0001), PLR (AUC = 0.603, *P* = 0.0049) and LMR (AUC = 0.685, *P* < 0.0001). The ability of NLR in predicting in-hospital death in the NSTEMI group was further compared with other common biomarkers of myocardial injury (such as AST, CK, LDH and TnI), and the results showed that NLR has the best predictive performance (Table 3 and Figures 1A,C). In contrast, it seemed that LMR (AUC = 0.661, *P* < 0.0001) showed better predictive ability than NLR (AUC = 0.621, *P* < 0.0001) in the STEMI group, but the AUC of both LMR and NLR were much lower than that of the NSTEMI group (Table 4 and Figures 1B,D). Therefore, NLR from BRE showed significant value in predicting in-hospital cardiac death in NSTEMI, deserving further exploration.

**Table 3:** The ROC curve and cut-off values of common ratios and markers of myocardial injury predicting in-hospital death in NSTEMI group.

**Variables**	**AUC**	**Sensitivity**	**Specificity**	**Youden index J**	**Cut-off value**	**95%CI**	***P*-value (z statistic)**
NLR	0.746	77.01%	67.72%	0.447	5.509	0.689–0.804	<0.001***
NMR	0.654	67.82%	58.07%	0.259	13.280	0.594–0.714	<0.001***
PLR	0.603	54.02%	73.04%	0.271	184.870	0.531–0.675	0.005**
LMR	0.685	70.11%	66.89%	0.370	2.412	0.620–0.749	<0.001**
LDH (U/L)	0.653	47.1%	75.3%	0.252	321.000	0.590–0.716	<0.001**
AST (U/L)	0.621	54.02%	67.80%	0.218	43.000	0.553–0.689	<0.001***
CK (U/L)	0.595	52.87%	68.22%	0.2109	258.000	0.526–0.663	0.007**
TnI (ng/ml)	0.594	44.83%	75.46%	0.203	2.690	0.526–0.663	0.007**

*P < 0.05; **P < 0.01; ***P < 0.001. NLR, neutrophil-to-lymphocyte ratio; NMR, neutrophil-monocyte ratio; PLR, platelet-lymphocyte ratio; LMR, lymphocyte-monocyte ratio; AST, aspartate aminotransferase; LDH, lactate dehydrogenase; CK, creatine kinase.

**Table 4:** The ROC curve and cut-off values of common ratios and markers of myocardial injury predicting in-hospital death in STEMI group.

**Variables**	**AUC**	**Sensitivity**	**Specificity**	**Youden index J**	**Cut-off value**	**95%CI**	***P*-value (z statistic)**
NLR	0.621	50.94%	71.63%	0.226	8.740	0.563–0.678	<0.001***
NMR	0.592	37.74%	79.15%	0.169	108.610	0.532–0.651	0.002**
PLR	0.512	39.62%	68.60%	0.082	195.370	0.451–0.572	0.705
LMR	0.661	66.98%	63.37%	0.304	2.400	0.603–0.719	<0.001***
LDH (U/L)	0.514	31.13%	74.98%	0.061	713.000	0.453–0.576	0.646
AST (U/L)	0.520	44.34%	64.80%	0.092	48.000	0.458–0.583	0.526
CK (U/L)	0.545	82.08%	26.98%	0.091	1629.000	0.486–0.605	0.136
TnI (ng/ml)	0.513	33.02%	75.80%	0.088	0.540	0.454–0.572	0.658

*P < 0.05; **P < 0.01; ***P < 0.001. NLR, neutrophil-to-lymphocyte ratio; NMR, neutrophil-monocyte ratio; PLR, platelet-lymphocyte ratio; LMR, lymphocyte-monocyte ratio; AST, aspartate aminotransferase; LDH, lactate dehydrogenase; CK, creatine kinase.

### NLR Is Associated With the Severity of AMI According to GRACE Score

NSTEMI and STEMI patients were divided into “low risk,” “middle risk” and “high risk” groups according to GRACE score (<109 score, 109–140 score, >140 score, respectively). In NSTEMI group, the quartiles of NLR in “low risk,” “middle risk” and “high risk” groups were 3.01(2.01, 4.33), 3.39(2.36, 5.10) and 5.49(3.44, 9.65) (*P* < 0.001). In STEMI group, the quartiles of NLR in “low risk,” “middle risk” and “high risk” groups were 5.08(3.07, 8.14), 5.17(2.98, 9.05) and 6.49(3.86, 11.68) (*P* < 0.001). Pearson analysis showed that NLR was related to GRACE score in STEMI group (*r* = 0.217, *P* < 0.001) and NSTEMI group (*r* = 0.264, *P* < 0.001). The high risk groups in STEMI and NSTEMI patients had the highest NLR, compared to the other two groups.

### NLR Is an Important Indicator Predicting Cardiac Death During Hospitalization in NSTEMI Patients

**Figure 2:**
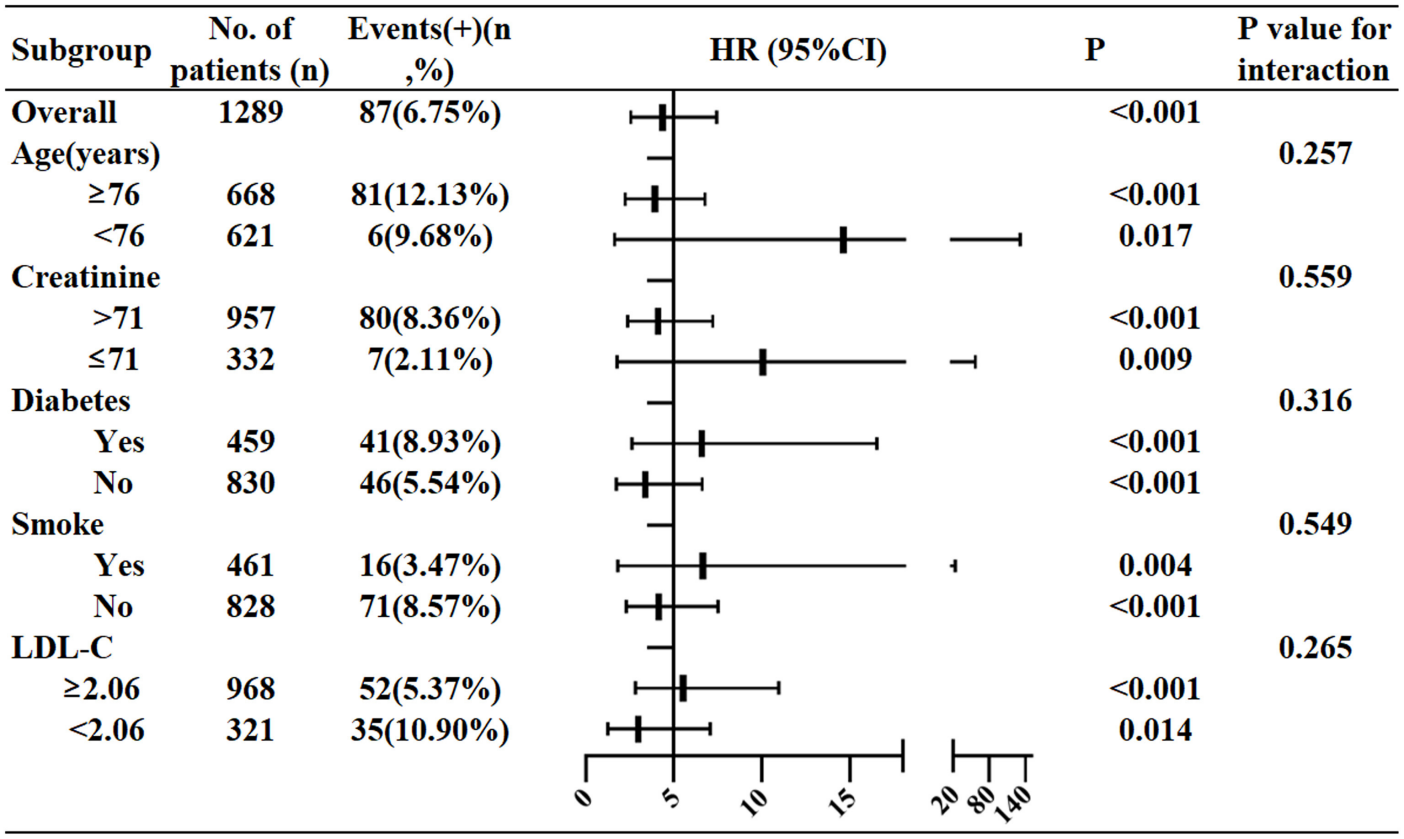
Hazard ratios of the NLR for predicting in-hospital death in NSTEMI group in the subgroup analysis.

The optimal cut-off value of NLR in the NSTEMI group was determined by Youden index from the ROC results. NLR at 5.509 was the best threshold with the optimal sensitivity (77.01%) and specificity (67.72%). Then the univariate logistic regression showed that the patients with NLR > 5.509 had higher hazard risk of death (HR7.028; 95%CI 4.204–11.749; *P* < 0.001). After adjusting variables (age, sex, diabetes history, smoking history, LDL-C and Cr), the adjusted HR was 4.356 (Figure 2) and that meant that the patients with NLR > 5.509 had a 4.356-fold higher risk of mortality than those with NLR ≤ 5.509.

### Stratification Analysis of NLR at Cut-Off Value in NSTEMI Patients

In order to assess the performance of NLR 5.509 as the optimal threshold in NSTEMI, subgroup stratification analysis was performed (Figure 2). After stratifying age (<76 or ≥76), creatinine ( ≤ 71 or >71), diabetes history (yes or no), smoking history (yes or no), LDL-C (<2.06 or ≥2.06), the predicting values of NLR 5.509 remained consistent. When stratified by age, the in-hospital mortality of NLR > 5.509 to NLR ≤ 5.509 in patients aged <76 was 14.611-fold (95%CI 1.623–131.494, *P* = 0.017), much higher than the ratio in patients aged ≥ 76. Apart from this, for patients with creatinine level ≤ 71, the in-hospital death risk in high NLR group was 10.065-fold higher than low NLR group (95%CI 1.761–57.514, *P* = 0.009), while the HR was only 4.117 in patients with creatinine level > 71 (95%CI 2.352–7.206, *P* < 0.001). For patients with diabetes, the death risk in the NLR group was 6.586-fold higher than in the low NLR group (95%CI 2.625–16.522, *P* < 0.001), while the HR was 3.375 in patients without diabetes (95%CI 1.724–6.605, *P* < 0.001). For smoking patients, the death risk in the NLR group was 6.646-fold higher than in the low NLR group (95%CI 1.807–24.436, *P* = 0.004), while the HR was 4.415 in non-smoking patients (95%CI 2.291–7.499, *P* < 0.001). For patients with LDL-C level ≥ 2.06, the in-hospital death risk in high NLR group was 5.526-fold higher than in low NLR group (95%CI 2.793–10.935, *P* < 0.001), while the HR was only 2.967 in patients with LDL-C level <2.06 (95%CI 1.249–7.048, *P* = 0.014).

## Discussion

In this retrospective study of 2,618 patients, we compared the prognostic efficacy of NLR and other BRE ratios (such as NMR, PLR, and LMR) in NSTEMI and STEMI. We mainly found that NLR was a better predictor of in-hospital death than other common BRE ratios. In addition, NLR showed the best performance in predicting cardiac death during hospital in NSTEMI patients rather than STEMI group, and the optimal threshold was 5.509. NLR was also related to the severity of AMI based on the stratification of GRACE score. Therefore, NLR > 5.509 may be a potential predictor of adverse outcome in NSTEMI patients.

Due to the high efficiency and low cost of BRE in emergency, it is necessary to keep exploring the significant roles of blood cell counts. White blood cells, including neutrophils and lymphocytes, are usually produced by the bone marrow. Within 24 h after entering the bloodstream, they penetrate the vascular wall in a deformable movement and enter the surrounding tissues. Because neutrophils have strong chemotactic and phagocytic functions, necrotic myocardial tissue can be engulfed by neutrophils. Neutrophils can remain 6–8 h in the blood after entering the blood stream from the bone marrow, and survive for 2–3 days in connective tissues. Under inflammatory stimulation of the body, the excessive production and release of neutrophils can promote the formation and development of atherosclerosis. Neutrophils will initiate the apoptotic program, form apoptotic fragments, participate in the formation of lipid pools, and promote the formation of thin-cap fibroatheroma (TCFA) (4). Tye 1 MI, the most important and common MI, is characterized as acute coronary atherosclerotic plaque disruption, usually caused by the rupture of TCFA (12). The neutrophils release neutrophil extracellular traps (NETs), including a variety of proteases, collagenases, etc., so that TCFA is easy to rupture and cause AMI. Within 24 h of MI, a large number of damage-related molecular patterns (DAMPs) produced by necrotic myocardium in the infarct area, through binding to the TLR family receptors on the surface of neutrophils, further induce the production of a large number of pro-inflammatory neutrophils (N1) with the proportion of 98%, and release a large quantity of inflammatory factors and reactive oxygen species, which promote myocardial damage in the infarcted area (13). Therefore, in the early stage after MI, neutrophils will increase significantly, which was consistent with our result. Under the stress state of MI, the activation of the neuroendocrine system leads to increased levels of catecholamines and glucocorticoids in the body, promotes the apoptosis of lymphocytes, and inhibits the proliferation and differentiation of lymphocytes, resulting in a decrease in peripheral blood lymphocyte count (14, 15). Therefore, the decrease in lymphocyte count is closely related to the severity of MI. In our study, the lymphocytes count showed a significant decline in death groups of both STEMI and NSTEMI. Blum et al. (16) found that the level of CD4+ T cells and the ratio of CD4+/CD8+ T cells in patients with MI were decreased, which was related to impaired heart function. T lymphocytes participate in repairing the myocardium (17) and B lymphocytes may promote myocardial fibrosis after myocardial infarction (18). Neutrophil count represents inflammation and lymphocyte count represents inflammatory response and stress state in the body. NLR may systematically and accurately reflect the degree of inflammation and stress. Then we used the GRACE score to stratify the risk of STEMI and NSTEMI, and found that the NLR of the high-risk group was significantly higher than that of the low-risk group, and NLR was significantly positively correlated with the severity of STEMI and NSTEMI.

NLR could predict in-hospital death after AMI, the ability of which is better in NSTEMI patients than in STEMI. In our study, comparisons among the common inflammatory markers from BRE were performed. The predictive efficacy of NLR in the NSTEMI group was higher than NMR, PLR and LMR, and also higher than that in the STEMI group. The early manifestations of coronary artery disease (CAD) were increased leukocytes and neutrophils and decreased lymphocytes. The higher the neutrophil count and the lower the lymphocyte count, the higher the level of inflammation and stress in the body, and the more serious the myocardial injury (19, 20). The blood samples of STEMI patients were usually from the first hours after AMI onset, but when NSTEMI patients felt sick and went to hospitals, the time after onset was longer than that of STEMI. That meant the onset time of STEMI was clear, but NSTEMI was not. Under inflammatory stress, it takes several hours for neutrophils to be recruited and released. When the patients were admitted to the hospital for blood collection, neutrophils count had not risen significantly. That may be one of the important reasons why NLR showed better performance in NSTEMI group.

There were some studies reporting the relationship between NLR and AMI. Previous studies with small sample sizes also showed NLR could predict adverse outcomes in AMI (21). A retrospective study with 107 patients assessed the potential of NLR predicting MACE after AMI and found high NLR (≥6.07) was an important predictor for the worst outcome (22), but this study did not distinguish STEMI and NSTEMI, and the sample size was too small. Another study retrospectively analyzed 806 AMI patients with left main and/or three-vessel disease, and found NLR was an effective predictor of 2-year all-cause death (23). Ching-Hui Sia et al. retrospectively analyzed the data from 289 post-AMI patients with left ventricular thrombosis and found that NLR was an important predictor of thrombus resolution after AMI (24). A retrospective study including 613 STEMI patients found NLR was positively associated with residual SYNTAX score (rSS), and NLR > 2.59 was an independent predictor of increased rSS (25). These results all support NLR as having an effective predicting ability of poor outcomes and severe conditions of coronary arteries. However, due to the distinction of definitions of outcomes in different studies, there was a difference in determining the optimal cut-off values of different outcomes. In our study, the optimal threshold of NLR for predicting in-hospital death after NSTEMI was 5.509. However, there was also an opinion opposite to the above results. David Hong et al. studied the relationship between NLR and AMI in 309 patients and they assessed the infarct size by cardiac magnetic resonance imaging. After adjusting variables such as age, sex, diabetes mellitus etc., NLR ≥ 3.88 cannot predict the high incidence of all-cause death (*P* = 0.101) and MI (*P* = 0.088) (21). By contrast, our results showed that after adjusting variables (age, sex, diabetes history, smoking history, LDL-C and Cr), the in-hospital death risk of patients with NLR>5.509 was 4.356-fold higher than those with NLR ≤ 5.509 (*P* < 0.001).

There are also previous studies reporting the roles of NLR in other cardiovascular diseases. One cross-sectional study showed that frailty was positively associated with the NLR (*r* = 0.169) in elderly CAD patients (26). Alexander J Fowler et al. performed a prospective clinical trial in patients with CAD receiving cardiac catheterization and found that the severity of the coronary artery lesions was positively correlated with NLR (27). Tse et al. found Quartile 4 of NLR was a significant indicator predicting all-cause mortality (OR2.36, *P* = 0.018) in patients with heart failure by multi-modality machine learning approaches, but not significant in predicting transient ischemic attack (TIA)/stroke and new-onset atrial fibrillation (28). In a retrospective study with 197 heart failure with reduced ejection fraction (HFrEF) patients, the optimal cut-off value of NLR for predicting 6-month mortality in HFrEF patients was 7.5 with 50% sensitivity and 91.7% specificity (OR1.259, *P* < 0.001) (5). These studies all indicated the important role of NLR in heart diseases.

There were some limitations in this study. First, the long-term prognosis of patients has not been observed, and it limited the analysis of NLR on predicting long-term mortality after AMI. Second, as a single-center and retrospective study, bias caused by confounding factors was a prominent problem, but methods such as stratified analysis and variate logistic regression analysis were used to eliminate the interference of confounding factors. The strengths of this study were that based on the cohort including 2618 AMI patients, we found NLR had better predicting ability in NSTEMI group, instead of STEMI group. We also demonstrated that NLR was positively associated with GRACE score, potentially guiding the stratification of AMI.

## Conclusions

In conclusion, NLR had a good ability in predicting in-hospital death after NSTEMI and had the limited predictive ability in STEMI. NLR was related to the severity of AMI. This study provided a new evidence for the role of NLR in the risk classification of AMI.

## Data Availability Statement

The raw data supporting the conclusions of this article will be made available by the authors, without undue reservation.

## Ethics Statement

The studies involving human participants were reviewed and approved by IEC for Clinical Research of Zhongda Hospital, Affiliated to Southeast University. Written informed consent for participation was not required for this study in accordance with the national legislation and the institutional requirements.

## Author Contributions

GM, YY, and ZJ: conception and design. ZJ and GL: data collection, data analysis and interpretation, and article writing. JG, RZ, YS, AC, YQ, WZ, YY, and JL: critical revision of the article. All authors make contributions to the article and approve the submission and publication of this manuscript.

## Funding

This study was funded by the National Natural Science Foundation of China (granted number 82070295) and Jiangsu Provincial Key Medical Discipline (Laboratory ZDXKA2016023).

## Conflict of Interest

The authors declare that the research was conducted in the absence of any commercial or financial relationships that could be construed as a potential conflict of interest.

## Publisher's Note

All claims expressed in this article are solely those of the authors and do not necessarily represent those of their affiliated organizations, or those of the publisher, the editors and the reviewers. Any product that may be evaluated in this article, or claim that may be made by its manufacturer, is not guaranteed or endorsed by the publisher.
