# Supplementary material for: The Neutrophil-to-Lymphocyte Ratio Is an Important Indicator Predicting In-Hospital Death in AMI Patients
Source: Front Cardiovasc Med. 2021 Sep 20;8:706852. doi: 10.3389/fcvm.2021.706852 (PMC8488114; doi:10.3389/fcvm.2021.706852)
Supplement: Supplementary file 1 [file Table_1.DOCX]

Table S1 Amount of missing data per variable in NSTEMI group. Alamine aminotransferase (ALT), Aspartate aminotransferase (AST), Lactate dehydrogenase (LDH), Low-density lipoprotein (LDLC), HIgh-density lipoprotein (HDLC), Blood urea nitrogen (BUN), Uric acid (UA), Total cholesterol (TC), Albumin (ALB), Myoglobin (Mb), Creatine kinase (CK), Glucose (GLU), White blood cells (WBC), Mean corpusular volume (MCV), Mean corpsular hemoglobin (MCH), Mean corpsular hemoglobin concentration (MCHC), Mean platelet volume (MPV), Hematocrit (Hct), Hemoglobin (Hb), Glycosylated hemoglobin (HbAC), Total bilirubin (TBil), Total bile acid (TBA), Total protein (TP), Triglyceride (TG), Direct bilirubin (DBil), Alkaline phosphatase (ALP), Cholinesterase (ChE), Lipoprotein a (Lpa), Brain natriuretic peptide (BNP), High sensitivity C reactive protein (hsCRP), Apolipoprotein B (apoB), Apolipoprotein A (apoA), Indirect bilirubin (IBil), Platelet-large cell rate (PLCR), Red blood cell volume distribution width coefficient variation (RDWCV), Red blood cell volume distribution width standard deviation (RDWSD), Platelet (PLT), Thyroid Stimulating Hormone (TSH), Free thyroxine 3 (FT3), Free thyroxine 4 (FT4), prothrombin time (PT), International normalized ratio (INR), Prothrombin activity (PTA),  Thrombin time (TT), Activated partial thromboplastin time (APTT), Fibrinogen Degradation Products (FDP), Fibrinogen (Fbg) , Cystatin C (CysC).

| **Variables** | **Missing, N** | **Missing, %** |
| --- | --- | --- |
| ALT | 65 | 5.0 |
| AST | 62 | 4.7 |
| LDH | 62 | 4.7 |
| LDLC | 98 | 7.4 |
| HDLC | 98 | 7.4 |
| BUN | 26 | 2.0 |
| UA | 21 | 1.6 |
| cTnI | 258 | 19.4 |
| TC | 67 | 5.0 |
| TBil | 85 | 6.4 |
| TBA | 87 | 6.5 |
| TP | 65 | 4.9 |
| TG | 67 | 5.0 |
| ALB | 65 | 4.9 |
| DBil | 65 | 4.9 |
| ALP | 65 | 4.9 |
| Mb | 800 | 60.2 |
| Creatine | 21 | 1.6 |
| CK | 117 | 8.8 |
| ChE | 65 | 4.9 |
| CysC | 676 | 50.9 |
| Lpa | 98 | 7.4 |
| BNP | 953 | 71.7 |
| GLU | 21 | 1.6 |
| hsCRP | 865 | 65.1 |
| apoB | 98 | 7.4 |
| apoA | 98 | 7.4 |
| HbA1c | 946 | 71.2 |
| IBil | 498 | 37.5 |
| neutrophil | 88 | 6.6 |
| N% | 88 | 6.6 |
| monocyte | 88 | 6.6 |
| M% | 88 | 6.6 |
| Baso | 87 | 6.5 |
| B% | 87 | 6.5 |
| Eos | 87 | 6.5 |
| E% | 87 | 6.5 |
| PLCR | 87 | 6.5 |
| MCV | 87 | 6.5 |
| MCH | 87 | 6.5 |
| MCHC | 87 | 6.5 |
| MPV | 96 | 7.2 |
| WBC | 87 | 6.5 |
| Lymph | 88 | 6.6 |
| L% | 88 | 6.6 |
| RDWCV | 87 | 6.5 |
| RDWSD | 87 | 6.5 |
| Hct | 87 | 6.5 |
| PLT | 87 | 6.5 |
| PDW | 96 | 7.2 |
| PCT | 96 | 7.2 |
| Hb | 87 | 6.5 |
| TSH | 604 | 45.4 |
| FT3 | 604 | 45.4 |
| FT4 | 604 | 45.4 |
| PT | 238 | 17.9 |
| Ddimer | 226 | 17.0 |
| INR | 238 | 17.9 |
| PTA | 711 | 53.5 |
| TT | 252 | 19.0 |
| APTT | 250 | 18.8 |
| FDP | 259 | 19.5 |
| Fbg | 241 | 18.1 |
