# Supplementary material for: The Neutrophil-to-Lymphocyte Ratio Is an Important Indicator Predicting In-Hospital Death in AMI Patients
Source: Front Cardiovasc Med. 2021 Sep 20;8:706852. doi: 10.3389/fcvm.2021.706852 (PMC8488114; doi:10.3389/fcvm.2021.706852)
Supplement: Supplementary file 3 [file Table_3.docx]

Table S3 Basic characteristics of echocardiography, medical history and concomitant medication in patients. If the continuous data fit a normal distribution, it was described by X±SD, otherwise, it was described by median and quartile(25%, 75%).

| Characteristics | STEMI | | P value | NSTEMI | | P value |
| --- | --- | --- | --- | --- | --- | --- |
|  | Without events  (n=1223) | With events  (n=106) |  | Without events  (n=1202) | With events  (n=87) |  |
| Echocardiography | | | | | | |
| LVEF | 0.55±0.12 | 0.47±0.19 | 0.001** | 0.60±0.13 | 0.50±0.16 | <0.001*** |
| Medical history | | | | | | |
| HBP | 1223(733) | 106(72) | 0.106 | 1202(903) | 87(71) | 0.174 |
| Diabete | 1223(353) | 106(47) | 0.001** | 1202(418) | 87(41) | 0.020* |
| CKD | 1223(73) | 106(15) | 0.001** | 1202(130) | 87(27) | <0.001*** |
| Concomitant medication | | | | | | |
| Antidiabetic drugs | 1219(221) | 102(15) | 0.386 | 1202(284) | 86(16) | 0.287 |
| Hypotensive drugs | 1219(882) | 102(26) | <0.001*** | 1202(894) | 86(41) | <0.001*** |
| Lipid-lowering drugs | 1219(1151) | 102(67) | <0.001*** | 1202(1158) | 86(61) | <0.001*** |
| Aspirin | 1219(1147) | 102(72) | <0.001*** | 1202(1166) | 86(67) | <0.001*** |
| Diuretics | 1219(503) | 102(23) | <0.001*** | 1202(500) | 86(23) | 0.007** |
| β-blockers | 1219(1015) | 102(41) | <0.001*** | 1202(998) | 86(49) | <0.001*** |

*P<0.05; **P<0.01; ***P<0.001. Left ventricular ejected fraction (LVEF), Hypertension (HBP), Chronic kidney disease (CKD).
